# Geographical and Ethnic Distributions of the MTHFR C677T, A1298C and MTRR A66G Gene Polymorphisms in Chinese Populations: A Meta-Analysis

**DOI:** 10.1371/journal.pone.0152414

**Published:** 2016-04-18

**Authors:** Xingmin Wang, Jinjian Fu, Qianxi Li, Dingyuan Zeng

**Affiliations:** Department of Gynecology, Liuzhou Municipal Maternity and Child Healthcare Hospital, Liuzhou, Guangxi, China; Duke Cancer Institute, UNITED STATES

## Abstract

**Background:**

The geographical and ethnic distributions of the polymorphic methylenetetrahydrofolate reductase (MTHFR) mutations (C677T and A1298C) and methionine synthase reductase (MTRR) mutation (A66G) remain heterogeneous in China. The goal of this study was to estimate the pooled frequencies of the alleles and associated genotypes of these gene polymorphisms among healthy populations in Mainland China.

**Objective and Methods:**

We systematically reviewed published epidemiological studies on the distributions of 3 genetic variants in Chinese healthy populations living in Mainland China through a meta-analysis. The relevant electronic databases were searched. All of the raw data of the eligible citations were extracted. The frequency estimates were stratified by geography, ethnicity and sex.

**Results:**

Sixty-six studies were identified with a total of 92277 study participants. The meta-analysis revealed that the frequencies of the MTHFR C677T, A1298C, and MTRR A66G gene polymorphisms varied significantly between different ethnic groups and along geographical gradients. The frequencies of the 677T allele and 677TT genotype increased along the southern-central-northern direction across Mainland China (all *P*values≤0.001). The frequencies of the 1298C, 1298CC, 66G and 66GG genotypes decreased along the south-central-north direction across the country (all *P*values≤0.001).

**Conclusions:**

Our meta-analysis strongly indicates significant geographical and ethnic variations in the frequencies of the C677T, A1298C, and A66G gene polymorphisms in the folate metabolism pathway among Chinese populations.

## Introduction

Multiple epidemiological studies have demonstrated that homocysteine is an important biomarker with biological functions in the folate metabolism pathway. Hyperhomocysteinemia (HHCY) is a medical health problem characterized by elevated homocysteine concentrations in the plasma that has been identified as a key pathophysiological risk factor for a series of adverse events, including neural-tube defects, vascular dementia, pregnancy complications, cancers and psychiatric disorders [1–4]. Previous studies have revealed that the regulation of the plasma levels of homocysteine are quite complex and involve both environmental factors (such as folate acid and vitamin B_12_ intake) and hereditary components [5]. However, how a number of genes and hereditary determinants might contribute to HHCY remains unclear. Mutations in some key genes encoding homocysteine-metabolizing enzymes, such as methylenetetrahydrofolate reductase (MTHFR) C677T and A1298C and methionine synthase reductase (MTRR) A66G, may contribute to the risk of the development of hyperhomocysteinemia and thus leas to clinical disorders [6].

The enzyme MTHFR catalyzes the conversion of 5,10-methylenetetrahydrofolate to 5-methyletetrahydrofolate, which is the carbon donor for the methylation of homocysteine to methionine [7]. The C677T polymorphism is a point mutation at position 677 of the MTHFR gene that causes the substitution of alanine with valine, which leads to a reduction in enzyme activity and causes mild to moderate hyperhomocysteinemia and reduces plasma folate levels. Genome-wide association studies (GWASs) have confirmed the association between the MTHFR C677T genotype and homocysteine levels in healthy populations [8]. Along with those investigations, several studies have proposed that double 677CT/1298AC heterozygosity can result in a reduction in enzymatic activity that represents an important risk factor for congenital anomalies, particularly in patients with low blood folate and vitamin B_12_ concentrations [9].

The frequency distributions of MTHFR and MTRR polymorphisms, especially C677T, vary substantially between different regional and ethnic groups. For example, the frequency of the 677T allele has been found to be highest in north India (16.7%) and lowest was in east India (1.1%). Moreover, the highest frequency of the 677TT genotype has been found in the Rajput population (7.8%), and this genotype is absent in the Kom, Meitei, Paite, Thadou, Kabui, Munda, Oraon and Naikda population groups in India [10]. A number of studies have investigated the C677T and A1298C in MTHFR and A66G in MTRR polymorphisms in different ethnic and geographical regions in Chinese general populations, however, the results have been irreproducible and inconclusive [11].

Accurate information about the geographical and ethnic distributions of the alleles and associated genotypes of MTHFR and MTRR in Mainland China will enable the design of proper interventions (e.g., folic acid supplementation) in the general population to reduce the rates of some medical diseases [12].

We conducted this comprehensive meta-analysis that integrated multiple studies to provide an overall assessment of the key polymorphisms in the major folate pathway genes among general Chinese populations. Sex-stratified and northern-central-southern gradients in the heterozygosities and allele frequencies were also assessed.

## Materials and Methods

### Literature database

The following major electronic literature databases were searched in September 2015 without language restrictions: PubMed, the Chinese National Knowledge Infrastructure (CNKI), the Chinese Wanfang Database, the Chinese VIP Database, and Google Scholar. The keywords and medical subject headings “MTHFR”, “MTRR”, “methylenetetrahydrofolate reductase”, “methionine synthase reductase”, “folate pathway’, “polymorphisms” or “SNP”, and “Chinese” or “China” were used to scan for potentially relevant studies.

### Inclusion/exclusion criteria

The identified studies were eligible for inclusion if they met the following criteria: (1) published in Chinese or English, (2) the study participants were general Chinese populations who lived in Mainland China, (3) the evaluation of data related to any or all of the polymorphisms in MTHFR or MTRR in general Chinese populations, and (4) included data regarding genotype/allele counts of the C677T, A1298C and A66G polymorphisms among the population for the estimation of the frequencies and 95% confidence intervals (95% CIs). Studies were excluded if they met the following criteria: (1) reviews, lectures, editorials or correspondence letters, (2) the study participants were evaluated in terms of folate pathway gene polymorphisms associated with relevant diseases, (3) duplicated studies were eliminated, and only recently published studies were ultimately selected, and (4) if the same data were published in English and Chinese, only the English-language articles were included.

### Data extraction

Two authors (XM Wang and JJ Fu) independently extracted the following information: the first author’s name, publication year, investigated location, ethnic groups, age, sample source, sample size, genotyping method, genotype distribution, frequency, and 95% CI.

### Statistical analysis

The Hardy-Weinberg equilibrist (HWEs) were evaluated to determine whether the MTHFR C677T and A1298C and MTRR A66G genotype distributions were in genetic equilibrium (threshold set to 0.05) [13]. Meta-analyses of the prevalences of the allele frequencies (e.g., C677T: TT vs. total genotypes) and allele contrasts (e.g., C677T: T vs. total C+T) were performed using Stata statistical software version 13.0 (Stata corporation LP, College Station, Texas, USA). A random-effects model was used to account for the pooled relevant genotype frequencies and their corresponding 95% CIs. Stratified analyses were performed according to the northern-central-southern gradient, ethnicity, and sex. The heterogeneity among the studies was evaluated with the Cochrane chi-square (*x*^*2*^) and quantified with the *I*^*2*^ statistic [14–15]. Publication bias was evaluated using Begg’s funnel plots and Egger’s test (significant at *P*≤0.1) [16–17].

## Results

### Characteristics of the studies

**Fig 1:**
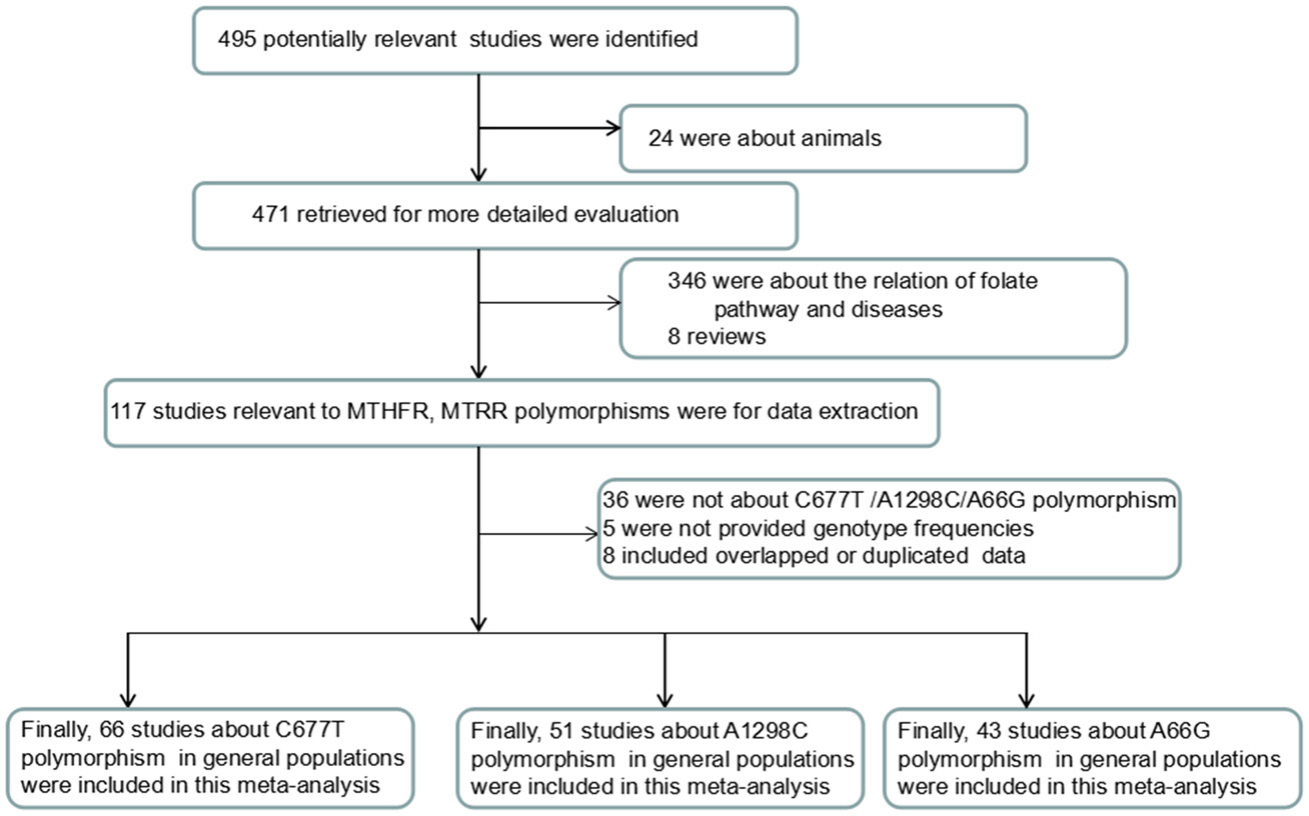
Flow chart of the study selection process.

In total, 495 articles were identified, of which 471 potentially relevant citations were included for further evaluation. Eventually, 68 articles (66 on the C677T, 51 on the A1298C, and 43 on the A66G polymorphisms) with a total of 92277 participants met the inclusion criteria [18–85] (Fig 1). The main characteristics of the studies on the MTHFR C677T and A1298C and MTRR A66G polymorphisms are listed in Tables 1–3, respectively. In the majority of the studies, buccal cells were obtained and tested with real-time polymerase chain reaction (RT-PCR); otherwise, blood samples were tested with restriction fragment length polymorphism (RFLP) analysis. The genotype frequencies indicated that all of the polymorphisms were in HWE in all of the samples.

**Table 1:** Distribution of the MTHFR C677T polymorphism among populations in China.

Author	Publicationyear	Location	Ethnicgroup	Age	Sample	Sample collection	Sample size(male/female)	Method	Genotype			Tallelic
									CC	CT	TT	
Yu JM	2000	mixed	mixed	not given	blood	convenient	200	RFLP	84	100	16	132
Pei LJ	2000	mixed	mixed	not given	blood	populational-based	277	RFLP	97	126	54	234
Zhu HP	1998	mixed	mixed	not given	blood	convenient	117	RFLP	50	50	17	84
Yang BH	2001	Anhui	Han	20–55	blood	convenient	55(30/25)	RFLP	19	21	15	51
Chen SQ	2002	Guangdong	Han	average 40	blood	convenient	143(68/75)	RFLP	90	50	3	56
Sun WP	2003	Shannxi	Han	37–78	blood	populational-based	96(58/38)	RFLP	26	53	17	87
Shen LP	2005	Guangxi	not given	23–34	blood	convenient	200(female)	RFLP	119	68	13	94
Xiao Y	2005	Guizhou	mixed	not given	blood	populational-based	317(138/179)	RFLP	221	90	6	102
Zhang CS	2005	Shandong	Han	44.7±7.5	blood	convenient	86(42/44)	RFLP	11	42	33	108
Li AF	2007	Henan	Han	56±9.8	blood	populational-based	500(274/226)	RFLP	163	173	164	501
Dai XJ	2008	Ningxia	mixed	18–22	blood	convenient	315(124/191)	RFLP	47	221	47	315
Mao FR	2008	mixed	mixed	not given	blood	populational-based	1015	RFLP	430	505	80	665
Chen F	2009	Henan	Han	35–76	blood	convenient	495(320/175)	RFLP	181	182	132	446
Shan KR	2009	Guizhou	Miao	not given	blood	populational-based	108	RFLP	88	17	3	23
Chen YX	2010	Shanxi	Han	25–35	blood	convenient	50(female)	RFLP	6	24	20	64
He XM	2010	mixed	Han	not given	blood	populational-based	1017(female)	RFLP	355	422	220	882
Jiang HO	2010	Hunan	Han	20–70	blood	populational-based	120	RFLP	64	41	15	71
Zhang QF	2010	Hainan	mixed	19–46	buccal cells	convenient	1008(female)	RT-PCR	559	310	139	588
Zhang L	2010	Guangxi	mixed	not given	blood	populational-based	1466(723/743)	RFLP	682	678	106	890
Lao HH	2011	Hainan	mixed	not given	buccal cells	populational-based	11437(female)	RT-PCR	6678	3741	1018	5777
Zhang Y	2012	Sichuan	Han	not given	buccal cells	populational-based	2573(female)	RT-PCR	1047	1171	355	1881
Wu HZ	2011	Anhui	Han	20–35	blood	populational-based	78(39/39)	RFLP	38	31	9	49
He YX	2012	Henan	Han	19–44	buccal cells	convenient	1093(female)	RT-PCR	198	493	402	1297
Yang Y	2012	Jiangsu	Han	27.0±4.4	buccal cells	convenient	2885(female)	RT-PCR	877	1378	630	2638
Cong YY	2012	Shandong	Han	29.4±7.7	buccal cells	convenient	1041(female)	RT-PCR	130	457	454	1365
Zhang YL	2012	Shandong	Han	28.7±5.8	buccal cells	convenient	825(female)	RT-PCR	138	398	289	976
Chen HB	2012	Shanxi	Han	not given	blood	convenient	63(31/32)	RFLP	10	31	22	75
Gao LJ	2012	Guangdong	Han	27.6±4.0	buccal cells	convenient	359(female)	RT-PCR	186	134	39	212
Du LL	2013	Guangxi	Zhuang	70–104	blood	populational-based	973(339/634)	RFLP	677	252	44	340
Yang BY	2013	mixed	Han	18–47	buccal cells	populational-based	15357(952/14405)	RT-PCR	4939	6791	3627	14045
Wang LN	2012	Xinjiang	mixed	19–65	blood	convenient	300(144/156)	RFLP	58	196	46	288
Xiu X	2013	Shandong	Han	19–40	buccal cells	convenient	2934(female)	RT-PCR	442	1354	1138	3630
Chen YX	2013	Shanxi	Han	22–73	blood	convenient	192(94/98)	RFLP	32	97	63	223
Wang WP	2013	Hubei	Han	28.2±3.3	buccal cells	convenient	2899(female)	RT-PCR	1069	1367	463	2293
Gao H	2013	Hubei	mixed	18–53	buccal cells	convenient	1902(female)	RT-PCR	696	902	304	1510
Wan LJ	2013	Yunnan	Han	27.5±4.0	buccal cells	convenient	297(female)	RT-PCR	116	139	42	223
Yan ZM	2013	Hainan	Han	27.2±5.3	buccal cells	convenient	1221(female)	RT-PCR	756	390	75	540
Zhang T	2013	Guizhou	minority	mixed	blood	populational-based	597(318/279)	RT-PCR	398	180	19	218
Huang GX	2013	Hainan	mixed	mixed	buccal cells	convenient	1841(female)	RT-PCR	1219	548	74	696
Luo XL	2014	Hubei	Han	27.3±5.2	buccal cells	convenient	1077(female)	RT-PCR	429	482	166	814
Wang FX	2014	Shannxi	Han	22–35	buccal cells	convenient	1508(female)	RT-PCR	918	249	341	931
Hao YY	2014	Xinjiang	mixed	mixed	buccal cells	convenient	210(female)	RT-PCR	83	86	41	168
Yan Q	2014	Shandong	Han	28.8±3.4	buccal cells	convenient	2670(female)	RT-PCR	497	1313	860	3033
Xing JF	2014	Henan	Han	28.2±4.2	buccal cells	convenient	425(female)	RT-PCR	57	207	158	523
Hu XW	2015	Hubei	Han	28.2±4.2	buccal cells	convenient	3963(female)	RT-PCR	1443	1845	675	3195
Jia XP	2015	Sichuan	Han	25.4±4.3	buccal cells	convenient	4865(female)	RT-PCR	1887	2243	735	3713
Huang QH	2015	Jiangsu	Han	26.5±4.3	buccal cells	convenient	348(female)	RT-PCR	99	192	58	308
Li JH	2015	Hebei	Han	27.3±4.9	buccal cells	convenient	1267(female)	RT-PCR	220	617	430	1477
Xiang CG	2015	Sichuan	Han	26.0±4.8	buccal cells	convenient	656(female)	RT-PCR	238	302	116	534
Jiang W	2014	Guangxi	mixed	28.0±4.5	buccal cells	convenient	948(female)	RT-PCR	572	315	61	437
Ouyang QQ	2014	Shandong	Han	22–39	blood	convenient	98(female)	RFLP	24	52	22	96
Chen XL	2014	Guangxi	Han	27.7±4.4	buccal cells	convenient	564(female)	RT-PCR	82	271	211	693
Ma LM	2015	Heilongjiang	Han	28.1±5.5	buccal cells	convenient	455(female)	RT-PCR	78	240	137	514
Tang HY	2014	Shandong	Han	27.7±3.8	buccal cells	convenient	787(female)	RT-PCR	107	373	307	987
Tian Y	2014	Jiangsu	Han	27.0±4.8	buccal cells	convenient	524(female)	RT-PCR	185	240	99	438
Lu GR	2014	Shandong	Han	28.5±5.0	buccal cells	convenient	1352(female)	RT-PCR	201	625	526	1677
Jiao FY	2014	Shandong	Han	28.2±4.2	buccal cells	convenient	529(female)	RT-PCR	93	261	175	611
Gao X	2014	Hebei	Han	28.3±4.3	buccal cells	convenient	860(female)	RT-PCR	158	429	273	829
Luo SQ	2015	Guangxi	Miao	not given	buccal cells	convenient	818(female)	RT-PCR	593	208	12	242
Yu YH	2015	Jilin	Han	28.5±4.3	buccal cells	convenient	2620(female)	RT-PCR	551	1253	816	2885
Li XX	2015	Jiangsu	Han	26.7±3.6	buccal cells	convenient	4008(female)	RT-PCR	1290	1984	734	1431
Wang SY	2015	Hunan	Han	26.7±4.6	buccal cells	convenient	1701(female)	RT-PCR	725	762	214	1190
Wu WQ	2015	Jiangsu	Han	26.4±4.5	buccal cells	convenient	644(female)	RT-PCR	189	308	147	602
Mao WC	2015	Guizhou	mixed	not given	buccal cells	convenient	1232(female)	RT-PCR	468	416	158	832
Cui HL	2015	Henan	Han	28.9±4.7	buccal cells	convenient	1253(female)	RT-PCR	201	542	510	1562
Liu XL	2014	Ningxia	Han	29.4±5.3	buccal cells	convenient	443(female)	RT-PCR	113	228	102	432

**Table 2:** Distribution of the MTHFR A1298C polymorphism among populations in China.

Author	Publicationyear	Location	Ethnicgroup	Age	Sample	Sample collection	Sample size (male/female)	Method	Genotype			C allelic
									AA	AC	CC	
Zhu HP	1998	mixed	mixed	not given	blood	convenient	117	RFLP	69	41	7	55
Sun WP	2003	Shannxi	Han	37–78	blood	populational-based	96(58/38)	RFLP	61	32	3	38
Xiao Y	2005	Guizhou	mixed	not given	blood	populational-based	317(138/179)	RFLP	100	184	33	250
Zhang CS	2005	Shandong	Han	44.7±7.5	blood	convenient	86(42/44)	RFLP	67	19	0	19
Mao FR	2008	mixed	mixed	not given	blood	populational-based	998	RFLP	391	385	222	829
Chen F	2009	Henan	Han	35–76	blood	convenient	495(320/175)	RFLP	387	105	3	111
He XM	2010	mixed	Han	not given	blood	populational-based	1017(female)	RFLP	649	322	46	414
Zhang QF	2010	Hainan	mixed	19–46	buccal cells	convenient	1008(female)	RT-PCR	585	342	81	504
Lao HH	2011	Hainan	mixed	not given	buccal cells	populational-based	11437(female)	RT-PCR	6481	4145	811	5767
Zhang Y	2012	Sichuan	Han	not given	buccal cells	populational-based	2573(female)	RT-PCR	1612	800	161	1122
Wu HZ	2011	Anhui	Han	20–35	blood	populational-based	78(39/39)	RFLP	46	30	2	34
He YX	2012	Henan	Han	19–44	buccal cells	convenient	1093(female)	RT-PCR	798	269	26	321
Yang Y	2012	Jiangsu	Han	27.0±4.4	buccal cells	convenient	2885(female)	RT-PCR	1993	791	101	993
Cong YY	2012	Shandong	Han	29.4±7.7	buccal cells	convenient	1041(female)	RT-PCR	822	204	15	234
Zhang YL	2012	Shandong	Han	28.7±5.8	buccal cells	convenient	825(female)	RT-PCR	627	178	20	218
Gao LJ	2012	Guangdong	Han	27.6±4.0	buccal cells	convenient	359(female)	RT-PCR	221	112	26	164
Yang BY	2013	mixed	Han	18–47	buccal cells	populational-based	13473	RT-PCR	9000	3944	529	5002
Xiu X	2013	Shandong	Han	19–40	buccal cells	convenient	2934(female)	RT-PCR	2224	672	38	744
Wang WP	2013	Hubei	Han	28.2±3.3	buccal cells	convenient	2899(female)	RT-PCR	1901	866	132	1130
Gao H	2013	Hubei	mixed	18–53	buccal cells	convenient	1902(female)	RT-PCR	1283	558	61	680
Wan LJ	2013	Yunnan	Han	27.5±4.0	buccal cells	convenient	297(female)	RT-PCR	194	95	8	111
Yan ZM	2013	Hainan	Han	27.2±5.3	buccal cells	convenient	1221(female)	RT-PCR	699	435	87	609
Zhang T	2013	Guizhou	minority	mixed	blood	populational-based	597(318/279)	RT-PCR	311	243	43	329
Wang P	2013	Xinjiang	mixed	Not given	buccal cells	convenient	300(female)	RT-PCR	204	91	5	101
Huang GX	2013	Hainan	mixed	mixed	buccal cells	convenient	1841(female)	RT-PCR	999	694	148	990
Luo XL	2014	Hubei	Han	27.3±5.2	buccal cells	convenient	1077(female)	RT-PCR	702	347	28	403
Wang FX	2014	Shannxi	Han	22–35	buccal cells	convenient	1508(female)	RT-PCR	542	912	54	1020
Hao YY	2014	Xinjiang	mixed	mixed	buccal cells	convenient	210(female)	RT-PCR	135	64	11	86
Yan Q	2014	Shandong	Han	28.8±3.4	buccal cells	convenient	2670(female)	RT-PCR	1936	685	49	783
Xing JF	2014	Henan	Han	28.2±4.2	buccal cells	convenient	425(female)	RT-PCR	316	102	4	110
Hu XW	2015	Hubei	NA	28.2±4.2	buccal cells	convenient	3963(female)	RT-PCR	2661	1168	134	1436
Jia XP	2015	Sichuan	Han	25.4±4.3	buccal cells	convenient	4865(female)	RT-PCR	3096	1555	214	1983
Huang QH	2015	Jiangsu	Han	26.5±4.3	buccal cells	convenient	348(female)	RT-PCR	231	106	10	126
Li JH	2015	Hebei	Han	27.3±4.9	buccal cells	convenient	1267(female)	RT-PCR	947	296	24	344
Xiang CG	2015	Sichuan	Han	26.0±4.8	buccal cells	convenient	656(female)	RT-PCR	428	205	23	251
Jiang W	2014	Guangxi	mixed	28.0±4.5	buccal cells	convenient	948(female)	RT-PCR	535	344	69	482
Chen XL	2014	Guangxi	Han	27.7±4.4	buccal cells	convenient	564(female)	RT-PCR	409	144	11	166
Ma LM	2015	Heilongjiang	Han	28.1±5.5	buccal cells	convenient	455(female)	RT-PCR	336	110	9	128
Tang HY	2014	Shandong	Han	27.7±3.8	buccal cells	convenient	787(female)	RT-PCR	607	168	12	192
Tian Y	2014	Jiangsu	Han	27.0±4.8	buccal cells	convenient	524(female)	RT-PCR	361	145	18	181
Lu GR	2014	Shandong	Han	28.5±5.0	buccal cells	convenient	1352(female)	RT-PCR	1027	297	28	353
Jiao FY	2014	Shandong	Han	28.2±4.2	buccal cells	convenient	529(female)	RT-PCR	400	117	12	141
Gao X	2014	Hebei	Han	28.3±4.3	buccal cells	convenient	860(female)	RT-PCR	624	216	20	435
Luo SQ	2015	Guangxi	Miao	NA	buccal cells	convenient	818(female)	RT-PCR	435	322	61	444
Li XX	2015	Jiangsu	Han	26.7±3.6	buccal cells	convenient	4008(female)	RT-PCR	2755	1152	101	768
Wang SY	2015	Hunan	Han	26.7±4.6	buccal cells	convenient	1701(female)	RT-PCR	1043	576	82	740
Wu WQ	2015	Jiangsu	Han	26.4±4.5	buccal cells	convenient	644(female)	RT-PCR	456	167	21	209
Mao WC	2015	Guizhou	mixed	not given	buccal cells	convenient	1232(female)	RT-PCR	803	380	49	478
Cui HL	2015	Henan	Han	28.9±4.7	buccal cells	convenient	1253(female)	RT-PCR	947	290	16	322
Liu XL	2014	Ningxia	Han	29.4±5.3	buccal cells	convenient	443(female)	RT-PCR	305	123	15	153
Yu YH	2015	Jilin	Han	28.5±4.3	buccal cells	convenient	2620(female)	RT-PCR	1912	639	69	777

**Table 3:** Distribution of the MTRR A66G polymorphism among populations in China.

Author	Publicationyear	Location	Ethnicgroup	Age	Sample	Sample collection	Sample size (male/female)	Method	Genotype			G allelic
									AA	AG	GG	
He XM	2010	mixed	Han	not given	blood	populational-based	1017(female)	RFLP	567	387	63	513
Zhang QF	2010	Hainan	mixed	19–46	buccal cells	convenient	1008(female)	RT-PCR	516	410	82	574
Lao HH	2011	Hainan	mixed	not given	buccal cells	populational-based	11437(female)	RT-PCR	5616	4763	1058	6879
Zhang Y	2012	Sichuan	Han	not given	buccal cells	populational-based	2573(female)	RT-PCR	1385	977	211	1399
He YX	2012	Henan	Han	19–44	buccal cells	convenient	1093(female)	RT-PCR	624	400	69	538
Yang Y	2012	Jiangsu	Han	27.0±4.4	buccal cells	convenient	2885(female)	RT-PCR	1642	1071	172	1415
Cong YY	2012	Shandong	Han	29.4±7.7	buccal cells	convenient	1041(female)	RT-PCR	610	381	50	481
Zhang YL	2012	Shandong	Han	28.7±5.8	buccal cells	convenient	825(female)	RT-PCR	451	325	49	423
Gao LJ	2012	Guangdong	Han	27.6±4.0	buccal cells	convenient	359(female)	RT-PCR	196	143	20	183
Yang BY	2013	mixed	Han	18–47	buccal cells	populational-based	15357(952/14405)	RT-PCR	8514	5834	1009	7852
Xiu X	2013	Shandong	Han	19–40	buccal cells	convenient	2934(female)	RT-PCR	1706	1060	168	1396
Wang WP	2013	Hubei	Han	28.2±3.3	buccal cells	convenient	2899(female)	RT-PCR	1650	1071	178	1427
Gao H	2013	Hubei	mixed	18–53	buccal cells	convenient	1902(female)	RT-PCR	1082	697	123	943
Wan LJ	2013	Yunnan	Han	27.5±4.0	buccal cells	convenient	297(female)	RT-PCR	172	106	19	144
Yan ZM	2013	Hainan	Han	27.2±5.3	buccal cells	convenient	1221(female)	RT-PCR	580	528	113	754
Lu XC	2013	Guangxi	Zhuang	mixed	buccal cells	convenient	300(female)	RT-PCR	83	117	20	157
Huang GX	2013	Hainan	mixed	mixed	buccal cells	convenient	1841(female)	RT-PCR	842	809	190	1189
Luo XL	2014	Hubei	Han	27.3±5.2	buccal cells	convenient	1077(female)	RT-PCR	579	429	69	563
Wang FX	2014	Shannxi	Han	22–35	buccal cells	convenient	1508(female)	RT-PCR	820	595	92	780
Hao YY	2014	Xinjiang	mixed	mixed	buccal cells	convenient	210(female)	RT-PCR	96	91	23	137
Yan Q	2014	Shandong	Han	28.8±3.4	buccal cells	convenient	2670(female)	RT-PCR	1459	1018	193	1404
Xing JF	2014	Henan	Han	28.2±4.2	buccal cells	convenient	425(female)	RT-PCR	241	162	19	200
Jia XP	2015	Sichuan	Han	25.4±4.3	buccal cells	convenient	4865(female)	RT-PCR	2748	1795	322	2439
Huang QH	2015	Jiangsu	Han	26.5±4.3	buccal cells	convenient	348(female)	RT-PCR	217	118	12	142
Li JH	2015	Hebei	Han	27.3±4.9	buccal cells	convenient	1267(female)	RT-PCR	705	496	66	628
Xiang CG	2015	Sichuan	Han	26.0±4.8	buccal cells	convenient	656(female)	RT-PCR	371	239	46	331
Jiang W	2014	Guangxi	mixed	28.0±4.5	buccal cells	convenient	948(female)	RT-PCR	501	376	71	518
Chen XL	2014	Guangxi	Han	27.7±4.4	buccal cells	convenient	564(female)	RT-PCR	324	209	31	271
Ma LM	2015	Heilongjiang	Han	28.1±5.5	buccal cells	convenient	455(female)	RT-PCR	245	184	26	236
Tang HY	2014	Shandong	Han	27.7±3.8	buccal cells	convenient	787(female)	RT-PCR	444	288	55	398
Tian Y	2014	Jiangsu	Han	27.0±4.8	buccal cells	convenient	524(female)	RT-PCR	298	191	35	261
Lu GR	2014	Shandong	Han	28.5±5.0	buccal cells	convenient	1352(female)	RT-PCR	779	498	75	648
Jiao FY	2014	Shandong	Han	28.2±4.2	buccal cells	convenient	529(female)	RT-PCR	285	200	44	288
Gao X	2014	Hebei	Han	28.3±4.3	buccal cells	convenient	860(female)	RT-PCR	460	334	66	530
Luo SQ	2015	Guangxi	Miao	not given	buccal cells	convenient	818(female)	RT-PCR	410	343	65	473
Yu YH	2015	Jilin	Han	28.5±4.3	buccal cells	convenient	2620(female)	RT-PCR	1479	977	164	1305
Li XX	2015	Jiangsu	Han	26.7±3.6	buccal cells	convenient	4008(female)	RT-PCR	2179	1543	286	1057
Wang SY	2015	Hunan	Han	26.7±4.6	buccal cells	convenient	1701(female)	RT-PCR	918	668	115	898
Wu WQ	2015	Jiangsu	Han	26.4±4.5	buccal cells	convenient	644(female)	RT-PCR	343	260	41	342
Mao WC	2015	Guizhou	mixed	not given	buccal cells	convenient	1232(female)	RT-PCR	718	437	76	590
Cui HL	2015	Henan	Han	28.9±4.7	buccal cells	convenient	1253(female)	RT-PCR	704	481	68	617
Liu XL	2014	Ningxia	Han	29.4±5.3	buccal cells	convenient	443(female)	RT-PCR	247	169	27	223
Hu XW	2015	Hubei	NA	28.2±4.2	buccal cells	convenient	3963(female)	RT-PCR	2247	1470	246	2962

### Pooled frequencies of the allele genotypes of the three gene polymorphisms in the Chinese general population

Table 4 illustrates the summarized national estimates of the 677TT and 677T frequencies among healthy populations from 1998 to 2015. Taking all populations together, the frequencies of the 677TT genotype and the 677T allele in the healthy Chinese population were 20% (18%-23%) and 42% (38%-45%), respectively (S1 and S2 Files). Overall, the combined estimated frequencies of the 1298CC genotype and the 1298C allele in the healthy Chinese population were 5% (4%-5%) and 20% (18%-22%), respectively(S3 and S4 Files). The average frequencies of the 66GG genotype and the 66G allele in the healthy Chinese population were 7% (6%-7%) and 26% (25%-28%), respectively(S5 and S6 Files).

**Table 4:** Summarized prevalence with 95% confidence intervals of genetic polymorphisms in the folate pathway among Chinese populations.

Polymorphisms	Genetic model	No. of studies	No. of provinces	No. of frequencies	Investigated number	Prevalence(95%CI)	Heterogeneity	
							I^2^(%)	P
MTHFR C677T	TT vs. total genotypes	66	23	18302	92277	0.20(0.18–0.23)	100.0	0.000
	Allele contrast	66	23	73823	184554	0.42(0.38–0.45)	100.0	0.000
MTHFR A1298C	CC vs. total genotypes	51	18	4051	85616	0.05(0.04–0.05)	100.0	0.000
	Allele contrast	51	18	33649	171232	0.20(0.18–0.22)	100.0	0.000
MTRR A66G	GG vs. total genotypes	43	16	5957	84636	0.07(0.06–0.07)	100.0	0.000
	Allele contrast	43	16	44508	169272	0.26(0.25–0.28)	100.0	0.000

### Geographical distributions of the three polymorphisms in the folate pathway

The allele and genotype frequencies of the three polymorphisms according to geographical region are given in Table 5. The genotype frequencies of the MTHFR C677T and 677T alleles and the 677TT genotype frequency exhibited increases in the southern-central-northern direction in Mainland China. The frequencies of the 677T allele and the 677TT genotype increased from lower values (5% and 17%, respectively) in Guangxi, to intermediate values (12% and 32%, respectively) in Anhui, to higher values (39% and 62%, respectively) in Shandong. Taken together, the frequencies of the 677TT genotype and the 677T allele along the geographical gradient were 7% (5%-8%) and 25% (23%-27%) in southern, 19% (16%-21%) and 41% (36%-45%) in central, and 28% (25%-31%) and 53% (51%-55%) in northern China, respectively. There were significant geographical gradients in the variations in the frequencies of the 677T allele and 677TT genotype (both *P* values≤0.001).

**Table 5:** Summarized prevalence with 95% confidence intervals of genetic polymorphisms in the folate pathway with geographical distribution among Chinese populations.

Polymorphisms	Latitude	Genetic model	No. of studies	No. of provinces	No. of frequencies	Investigated number	Prevalence(95%CI)	Heterogeneity	
								I^2^(%)	P
MTHFR C677T	southern China	TT vs. total genotypes	20	7	2131	27332	0.07(0.05–0.08)	100.0	0.000
		Allele contrast	20	7	13525	54664	0.25(0.23–0.27)	100.0	0.000
	central China	TT vs. total genotypes	19	6	7588	39205	0.19(0.16–0.21)	100.0	0.000
		Allele contrast	19	6	31075	78410	0.41(0.36–0.45)	100.0	0.000
	northern China	TT vs. total genotypes	27	10	8557	25569	0.28(0.25–0.31)	100.0	0.000
		Allele contrast	27	10	29134	51138	0.53(0.51–0.55)	100.0	0.000
MTHFR A1298C	southern China	CC vs. total genotypes	13	4	1705	26653	0.07(0.05–0.09)	100.0	0.000
		Allele contrast	13	4	12762	53306	0.28(0.24–0.31)	100.0	0.000
	central China	CC vs. total genotypes	19	7	1432	38936	0.04(0.03–0.04)	100.0	0.000
		Allele contrast	19	7	14182	77872	0.18(0.17–0.19)	100.0	0.000
	northern China	CC vs. total genotypes	19	7	692	19029	0.03(0.02–0.03)	100.0	0.000
		Allele contrast	19	7	5876	38054	0.17(0.16–0.19)	100.0	0.000
MTRR A66G	southern China	GG vs. total genotypes	4	3	402	4839	0.08(0.06–0.10)	100.0	0.000
		Allele contrast	4	3	2770	9678	0.29(0.28–0.30)	100.0	0.000
	central China	GG vs. total genotypes	19	6	2710	41728	0.06(0.06–0.07)	100.0	0.000
		Allele contrast	19	6	21068	83456	0.25(0.23–0.27)	100.0	0.000
	northern China	GG vs. total genotypes	20	7	1192	19759	0.06(0.05–0.06)	100.0	0.000
		Allele contrast	20	7	9810	39518	0.24(0.23–0.25)	100.0	0.000

The frequency of the MTHFR A1298C polymorphism exhibited the reverse trend; i.e., this frequency decreasing from southern to central to northern China. The pooled geographical gradient frequencies of the 1298C allele and 1298CC genotype were found to be 28% (24%-31%) and 7% (5%-9%) in southern, 18% (17%-19%) and 4% (3%-4%) in central, and 17% (16%-19%) and 3% (2%-3%) in northern China, respectively (Table 5). There were significant geographical gradients in the frequencies of the 1298C allele and 1298CC genotype (both *P* values≤0.001).

The mean frequencies of the MTRR 66G allele and 66GG genotype decreased from 29% (28%-30%) and 8% (6%-10%) in southern China, to 25% (23%-27%) and 6% (6%-7%) in central China, and 24% (23%-25%) and 6% (5%-6%) in northern China (Table 5) in a pattern similar to that observed in the gradients of the MTHFR 1298C allele and 1298CC genotype frequencies (both *P* values≤0.001).

### The frequencies of the MTHFR C677T, A1298C, and MTRR A66G polymorphisms by ethnicity

The allele and genotype distributions of MTHFR and MTRR by ethnicity are presented in Table 6. The distributions of the MTHFR 677T allele and the 677TT genotype exhibited ethnic variations (with both *P* values≤0.001). The 677T allele frequencies in the minority groups (e.g., Miao, Zhuang, She, Shui, etc.) and Chinese Han were 28% (25%-31%) and 45% (41%-49%), respectively. The 677TT genotype frequencies in the minority groups and Chinese Han were 5% (4%-6%) and 22% (20%-25%), respectively.

**Table 6:** Summarized prevalence with 95% confidence intervals of genetic polymorphisms in the folate pathway with ethnicity distribution among Chinese populations.

Polymorphisms	Ethnicity	Genetic model	No. of studies	No. of ethnic groups	No. of provinces	No. of frequencies	Investigated number	Prevalence(95%CI)	Heterogeneity	
									I^2^(%)	P
MTHFR C677T	Minority	TT vs. total genotypes	17	19	11	381	7559	0.05(0.04–0.06)	100.0	0.000
		Allele contrast	17	19	11	3390	15118	0.28(0.25–0.31)	100.0	0.000
	Han	TT vs. total genotypes	55	1	22	16973	78852	0.22(0.20–0.25)	100.0	0.000
		Allele contrast	55	1	22	66065	157704	0.45(0.41–0.49)	100.0	0.000
MTHFR A1298C	Minority	CC vs. total genotypes	10	8	4	368	4669	0.07(0.05–0.09)	100.0	0.000
		Allele contrast	10	8	4	2494	9338	0.26(0.23–0.30)	100.0	0.000
	Han	CC vs. total genotypes	44	1	17	3228	74454	0.04(0.03–0.05)	100.0	0.000
		Allele contrast	44	1	17	28205	148908	0.19(0.17–0.20)	100.0	0.000
MTRR A66G	Minority	GG vs. total genotypes	8	5	4	436	4792	0.10(0.08–0.12)	100.0	0.000
		Allele contrast	8	5	4	2918	9584	0.35(0.35–0.36)	100.0	0.000
	Han	GG vs. total genotypes	39	1	15	5210	75357	0.06(0.05–0.07)	100.0	0.000
		Allele contrast	39	1	15	38367	150714	0.25(0.24–0.26)	100.0	0.000

In contrast to C677T, the distribution of the A1298C polymorphism by ethnicity demonstrated the reverse trend: the 1298C allele was much more common among the minority groups [26%, (23%-30%)] than the Chinese Han [19% (17%-20%); *P* value≤0.001]. The 1298CC genotype exhibited similar variability with frequencies of 7% (5%-9%) in the minority groups and 4% (3%-5%) in the Chinese Han (*P* value ≤0.001).

The frequencies of the MTRR 66G allele and 66GG genotype varied by ethnic group and geographical location. The frequency of the 66G allele was slightly higher among the minority groups [35% (35%-36%)] compared with 25% (24%-26%) among the Chinese Han group (*P* value ≤0.001). The frequencies of the 66GG genotype were 10% (8%-12%) in the minority groups and 6% (5%-7%) in the Chinese Han group, which were similar to those of the MTHFR A1298C polymorphism (*P* value ≤0.001).

### The frequencies of MTHFR C677T, A1298C and MTRR A66G polymorphisms by sex

Table 7 provides the pooled frequencies of the variant alleles and genotypes of MTHFR C677T and A1298C and MTRR A66G according to sex. A total of 88255 samples with reported C677T polymorphisms were obtained. Based on all these samples, we did not find any difference between the males [19% (12%-25%)] and females [21% (19%-24%)] in terms of 677TTgenotype frequency.

**Table 7:** Summarized prevalence with 95% confidence intervals of genetic polymorphisms in the folate pathway with sex distribution among Chinese populations.

Polymorphisms	Gender	Genetic model	No. of studies	No. of provinces	No. of frequencies	Investigated number	Prevalence(95%CI)	Heterogeneity	
								I^2^(%)	P
MTHFR C677T	Male	TT vs. total genotypes	11	6	462	2507	0.19(0.12–0.25)	100.0	0.000
		Allele contrast	11	6	2380	5014	0.49(0.41–0.58)	100.0	0.000
	Female	TT vs. total genotypes	53	17	17311	85748	0.21(0.19–0.24)	100.0	0.000
		Allele contrast	53	17	69098	171496	0.44(0.40–0.47)	100.0	0.000
MTHFR A1298C	Female	CC vs. total genotypes	41	17	3733	82532	0.04(0.04–0.05)	100.0	0.000
		Allele contrast	41	17	31883	165064	0.19(0.18–0.21)	100.0	0.000
MTRR A66G	Female	GG vs. total genotypes	42	15	5907	84416	0.07(0.06–0.07)	100.0	0.000
		Allele contrast	42	15	44351	168832	0.26(0.25–0.27)	100.0	0.000

Only 41 studies reported the frequency of the MTHFR A1298C polymorphism and included 82532 females of reproductive age. The 1298C allele and 1298CC genotype frequencies in females were 19% (18%-21%) and 4% (4%-5%), respectively. Among the 43 articles that reported on the MTRR A66G polymorphism, 42 studies included 84416 females. The 66G allele and 66GG genotype frequencies in females were 26% (25%-27%) and 7% (6%-7%), respectively.

### Publication bias

Tables 4–7 presents information related to heterogeneity and publication bias. We noted significant heterogeneity within the studies and the subgroups (all *P* values were ≤0.001, *I*^*2*^ = 100.0).

## Discussion

Methylenetetrahydrofolate reductase (MTHFR) (C677T and A1298C) and methionine synthase reductase (MTRR) mutations (A66G) cause mild hyperhomocysteinemia and low folate level and are associated with several disorders. The geographical and ethnic distributions of these alleles and the associated genotypes are important to study worldwide.

The frequencies of the MTHFR C677T and A1298C and MTRR A66G polymorphism in 68 epidemiological studies covering 23 provinces in Mainland China were pooled and investigated in the present study. Currently, there is a lack of national data regarding the prevalences of gene polymorphisms in the folate metabolism pathway in healthy general populations in China. We documented distinctive geographical and ethnic variations in the frequencies of the C677T and A1298C polymorphisms of the MTHFR gene and the A66G polymorphisms of the MTRR gene among nation-wide samples in China.

Worldwide data have revealed that significant heterogeneities in the frequencies of the T allele and TT homozygosity exist in every population and even with racial groups. One investigations conducted in Texas reported that the frequency of the 677T was lowest among African-Americans (11.9%), followed by in Caucasians (32.7%) and Ashkenazi Jews (47.7%), and the highest frequency exists among the Hispanic population (47.9%) [86]. In the Chinese Han, the frequencies of the 677T allele have been found to be lowest in Hainan (24.0%) followed by Hubei (40.3%) and Jiangsu (43.5%), and the highest frequency has been observed in Shandong (63.1%) [51].

Population genetic comparisons provide an appropriate method for picturing geographical and ethnic variations and can suggest that environmental factors may exert selective pressures on genetic mutations. A north-to-south increase in the frequency of the 677T allele has been observed in Europe [87]. North-to-south increases in dietary folate intake have also been encountered in European populations [88]. Thus adequate folic acid intakes have presumed enabled increase in the MTHFR 677T frequency in these populations [89]. Economic and dietary habits might have played important roles in the spread of the 677T allele worldwide. For example, the frequency of the 677T allele is high in the USA with an average frequency of 36.2% in Texas [86]. Another study conducted in India observed the highest frequency of the 677T allele among the Sindhi population (23.8%). In contrast, the 677T allele is absent in the Kom, Thadou and Munda populations, and its average frequency is 10.1% across all 23 populations in India [10]. The low frequencies of the 677T allele among the tribal groups (i.e., the Kom, Thadou and Munda populations) may have been influenced by folate deficiencies because the majority of the population in India has a vegetarian diets that is low in vitamin B_12_ [10]. The populations of America carried higher frequencies of the 677T allele, which may be related to abundant nutritional statuses and particularly with folic acid and vitamin B_12_ supplementation, which are associated with low levels of homocysteinemia. Across all 23 of the studied provinces, we observed increases in the 677T allele and 677TT genotype frequencies in the southern-central-northern direction across Mainland China. Because high 677T allele and 677TT genotype frequencies were observed in the northern populations, we assumed that the folic acid intakes are greater in the northern populations than in the southern populations; however, the opposite pattern has been observed in nutritional studies. One such nutritional investigation revealed that the geometric mean of the blood folate concentration is lower in the northern populations than the southern populations [90].

Worldwide epidemiological data have revealed that the frequency of A1298C homozygosity varies from continent to continent. The frequencies of the 1298C allele range from 18% to 70% in East Asia, 17% to 44% in Asia, 24% to 40% in Europe, 0% to 15% in South America and 14.7% in North America [91]. The present data revealed variation in the frequency of the 1298C allele within China. In contrast to the distribution of 677T, the frequency of the 1298C allele was found to be the lowest in northern China [18% (17%-19%)], intermediate in central China [18% (17%-19%)], and highest in southern China [28% (24%-31%)]. The mean frequency of the 1298C allele was 20% (18%-22%).

Based on all 8 of the investigated minority ethnic populations (e.g., the She, Xibo, and Uygur), the minority ethnic populations seemed to carry greater 1298C allele frequencies than the Chinese Han population. Notably, the frequency of the 1298C allele has been reported to vary between different ethnic populations worldwide, and the lowest frequency has been found in Indians (10%) [92] followed by the Chinese (18.4%) [51] and Tamils (35%) [93], and the highest frequency has been observed in the Lebanese [94].

Although A1298C homozygotes do not exhibit elevated blood homocysteinemialevels, many investigations have revealed that compound heterozygotes for C677T/ A1298C may be at risk for hyperhomocysteinemia and low folate levels, which can contribute to many disorders, such as neutral tube defects [6] and abortions [95].

Because lifestyle and environmental factors, such as folate supplementation, vary across different ethnic populations and may influence the frequencies of the C677T and A1298C alleles, these factors cannot be ruled out when considering the influences of environmental-genetic interactions on the distributions of MTHFR gene polymorphisms.

Our pooled data revealed that the frequencies of the 66G allele and 66GG genotype exhibited variations across geographical gradients and ethnic populations. Globally, the distributions of the MTRR 66G allele and 66GG genotype frequencies also exhibit geographical and ethnic variations. For example, the frequencies of the 66G allele have been reported to be 58% in the Yadav, 62% in the Scheduled Castes, and 71% in the rural Sunni Muslim population in Uttar Pradesh in India [96,97]. Our study observed a 66GG genotype frequency of 7% across Mainland China, which is much lower than those in Brazil (23%), Australia (10%), and Ireland (17.5%) [98–100]. MTRR is involved in the homocysteine and folate metabolic pathway via its activation of methionine synthase via reductive methylation and is consequently a critical determinant of homocysteinemia levels [101]. Therefore, the MTRR A66G mutation may indirectly contribute to many medical disorders, such as neural tube defects and congenital heart disease [102], due to its key role in the folate metabolism pathway. However, due to limited sample sizes and the lower frequency of studies of the A66G polymorphisms in MTRR, no solid evidence has been found to relate the MTRR A66G variant with the risks of diseases. Long-term data and larger sample sizes are necessary to determine the real connections between the distribution of the A66G variant and the risks of diseases.

## Conclusions

In conclusion, our meta-analysis revealed significant geographical variations in the frequencies of the MTHFR C677T and A1298C and MTRR A66G polymorphisms in the folate metabolism pathway between different ethnic populations in China. Our findings provide an overall picture of these three genetic polymorphisms in the folate metabolism pathway among the general populations in Mainland China, and these evidence-based genomic data should be integrated into medical and public health practices.

## Supporting Information

S1 File The average frequencies of the 677TT genotype in the healthy Chinese population. (TXT)

S2 File The average frequencies of the 677T allele in the healthy Chinese population. (TXT)

S3 File The average frequencies of the 1298CC genotype in the healthy Chinese population. (TXT)

S4 File The average frequencies of the 1298C allele in the healthy Chinese population. (TXT)

S5 File The average frequencies of the 66GG genotype in the healthy Chinese population. (TXT)

S6 File The average frequencies of the 66G allele in the healthy Chinese population. (TXT)
